# Epigenetic silencing and genome dynamics determine the fate of giant virus endogenizations in *Acanthamoeba*

**DOI:** 10.1186/s12915-025-02280-1

**Published:** 2025-07-01

**Authors:** Cédric Blais, Morgan J. Colp, Luke A. Sarre, Alex de Mendoza, John M. Archibald

**Affiliations:** 1 Department of Biochemistry & Molecular Biology, Dalhousie University, Halifax, NS Canada; 2 Institute for Comparative Genomics, Dalhousie University, Halifax, NS Canada; 3 School of Biological and Behavioural Sciences, Queen Mary University of London, London, UK; 4 Centre for Epigenetics, Queen Mary University of London, London, UK

**Keywords:** Giant viruses, Endogenization, Epigenetic silencing, Transposable elements, Protists

## Abstract

**Background:**

Endogenized giant viruses are emerging as major contributors to the genome evolution of microbial eukaryotes, with both degraded and fully functional latent viruses being found integrated in diverse lineages. The mechanisms that determine the fate of viral integrants are poorly understood, however. *Acanthamoeba* is a unicellular eukaryote known for undergoing lateral gene transfer (LGT) with viruses. Here we have leveraged chromosome-scale assemblies of two strains of *Acanthamoeba*, Neff and C3, to investigate the genomic mechanisms that mediate the fate of viral integrations in eukaryotic genomes.

**Results:**

Viral integrations in the C3 and Neff genomes are largely non-overlapping and disproportionately found in sub-telomeric regions. Multiple partial copies of these insertions are found throughout the Neff genome, but they are not expressed, do not obviously encode functions associated with their own mobility, and are colonized by host mobile elements. Viral regions are hypermethylated and highly condensed, suggesting that the expression of recently acquired viral DNA is suppressed in heterochromatic regions.

**Conclusions:**

We propose a model for the trajectory of viral sequences in *Acanthamoeba*: (i) integration of DNA from giant viruses, (ii) epigenetic suppression of the viral DNAs, allowing them to persist in the genome, and (iii) deterioration of viral genomes by point mutation, mobile element colonization, and intra- and inter-chromosomal recombination. Viral integrations in *Acanthamoeba* spp. are transient and may not have long-lasting effects on the fitness of the amoeba. Our work highlights the importance of host genome dynamics and epigenetic silencing for understanding the evolution of endogenized viral elements.

**Supplementary Information:**

The online version contains supplementary material available at 10.1186/s12915-025-02280-1.

## Background

The acquisition and spread of mobile genetic elements are well-known drivers of genome evolution in eukaryotes. Large stretches of the genomes of plants, animals, and fungi consist of so-called junk DNA, which for the most part does not encode proteins used by the host cell and whose sequence evolves under relaxed selective constraints [1]. The role this junk DNA plays in the organism is hotly debated [2–4]. It is often considered a byproduct of the spread of selfish DNA, such as transposable elements and retroviruses, which replicate to their own advantage without benefitting the host. The factors that lead to the acquisition and retention or loss of virus-derived DNA remain poorly understood. Recent developments in comparative genomics show that we have much to learn from the realm of microbial eukaryotes, in which new classes of mobile and viral elements have been discovered. These elements form symbiotic and parasitic relationships with diverse lineages of eukaryotes and are part of a complex genomic ecosystem which provides numerous avenues for lateral gene transfer (LGT) within and between branches of the eukaryotic tree of life [5–7].

Members of the virus kingdom Bamfordvirae are emerging as a major source of foreign DNA in eukaryotic genomes [8]. The Bamfordvirae are incredibly diverse dsDNA viruses characterized by their use of double jelly roll major capsid proteins [9]. They notably include “giant” viruses, also known as nucleocytoplasmic large DNA viruses (NCLDVs). Comparative genomics suggests that the group has evolved viral gigantism multiple times [10], with the genomes of some NCLDVs being as large as 2.6 megabase-pairs (Mbp) [11]. NCLDVs have been shown to both donate genes and receive genes from eukaryotic genomes, in which they reside in various states of degradation [12, 13]. While most cultured representatives of NCLDVs are lytic, this is possibly because they are typically isolated by inoculating protist cultures and monitoring them for lysis [14]. Interestingly, a 300 kilobase-pair (kbp) endogenous mirusvirus—a chimeric relative of NCLDVs [15]—was recently found integrated in the genome of the thraustochytrid protist *Aurantiochytrium limacinum* (the organism also harbors a similarly sized, evolutionarily distinct episomal version of the same virus) [16]. Similarly, a 617 kbp integrated NCLDV was discovered in *Chlamydomonas reinhardtii* which could be induced to produce virions [17], raising the possibility of lysogenic cycles being widespread among NCLDVs. In addition to these endogenized giants, an extensive network of modular, interconnected, and highly diverse mobile elements has been shown to integrate into protist genomes [18]. It includes virophages, which are superparasitic viruses who target other viruses and may form mutualistic relationships protecting the cells in which they are integrated, and polinton-like viruses, which may alternate between mobile-element-like lifestyles and viral lifestyles [7] and may have evolved their own form of superparasitism [19].

The long-term effect of DNA viruses on the genome biology and evolution of eukaryotes remains unclear and is likely highly variable across the eukaryotic tree. Viral integrations do not necessarily make lasting contributions to host genome evolution. Viral insertions vary greatly from one genome to the next [20] and it can be difficult to distinguish “live” endogenized viruses which retain virulence from permanently integrated sequences co-evolving with the genome. While some protein-coding genes in eukaryotes appear to be the product of ancient LGT from viruses [21], for the most part viral contributions to eukaryotic genomes appear to be recent and transient [20, 22].

The genus of single-celled amoebae *Acanthamoeba* has been particularly fruitful for the study of the interplay between eukaryotes and Bamfordvirae. *Acanthamoeba* are hosts to a broad range of intracellular bacteria and viruses and have been referred to as melting pots of LGT [23]. The first recognized “giant” virus, *Mimivirus*, was isolated from *Acanthamoeba polyphaga* in 2003 [24], and *Acanthamoeba* has since proven to be permissive to an extremely broad range of NCLDVs [25]. This permissiveness has led it to be used to discover and isolate many new lineages of viruses [26], including virophages, which were found to co-occur with giant viruses in *Acanthamoeba* [27]. The *Acanthamoeba* genome bears the mark of these associations—hundreds of genes derived from viruses have been identified in several species of *Acanthamoeba*. For example, Maumus and Blanc identified several large, poorly expressed clusters of viral sequences in the original reference genome assembly of *Acanthamoeba* strain Neff [28] and provided evidence for collinearity between these viral integrations and some giant viral genomes [29]. However, the limited number of then-available Bamfordvirae genomes restricted the search for viral sequences, and the fragmented nature of the assembly prevented examination of the genomic context in which these transfers occurred. This made it difficult to draw inferences about the evolutionary trajectory of giant viral integrants in *Acanthamoeba* strains and related species.

We have performed a genome-wide investigation of viral DNA in two recently published chromosome-scale assemblies for *Acanthamoeba* strains Neff and C3 [30]. C3 is classified as a strain of *Acanthamoeba castellanii*, while Neff was recently proposed to belong to *A. terricola* rather than *A. castellanii* [31]. The high quality of these assemblies provides a unique opportunity to explore the genomic context of viral integrants in these two divergent strains, thereby allowing us to uncover factors that shape their acquisition and fate in a nuclear context. Our results suggest that epigenetic suppression of viral DNA expression is an important factor limiting deleterious impact of newly integrated viral DNAs, as is recombination and mobile-element-based disruption of intact viral genomes.

## Results

### The genomes of *Acanthamoeba* strains Neff and C3 both harbor hundreds of sequences from giant viruses

**Fig. 1:**
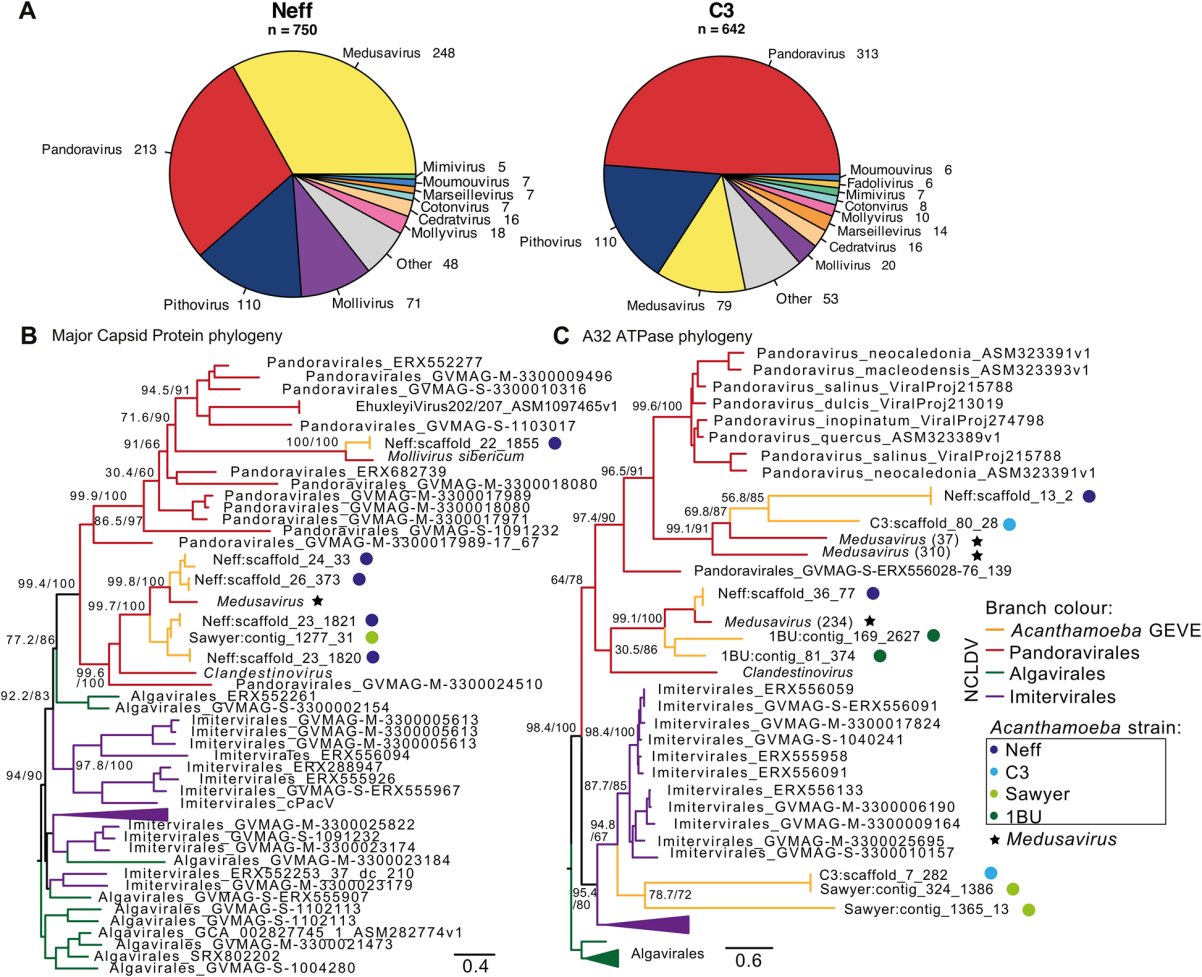
Phylogeny and taxonomic distribution of viral candidates in Neff and C3. **A** Taxonomic distribution of Neff and C3 viral candidates’ best hits to viruses. Taxa with less than five representatives were grouped together in “Other.” **B** Phylogeny of major capsid proteins found in Neff giant endogenous viral elements (GEVEs). **C** Phylogeny of A32 ATPases endogenized in Neff and C3. Both trees were built with IQ-Tree 2, with ultrafast bootstrap and aLTR values shown on branch nodes. The substitution model was auto-selected by IQ-Tree 2

**Fig. 2:**
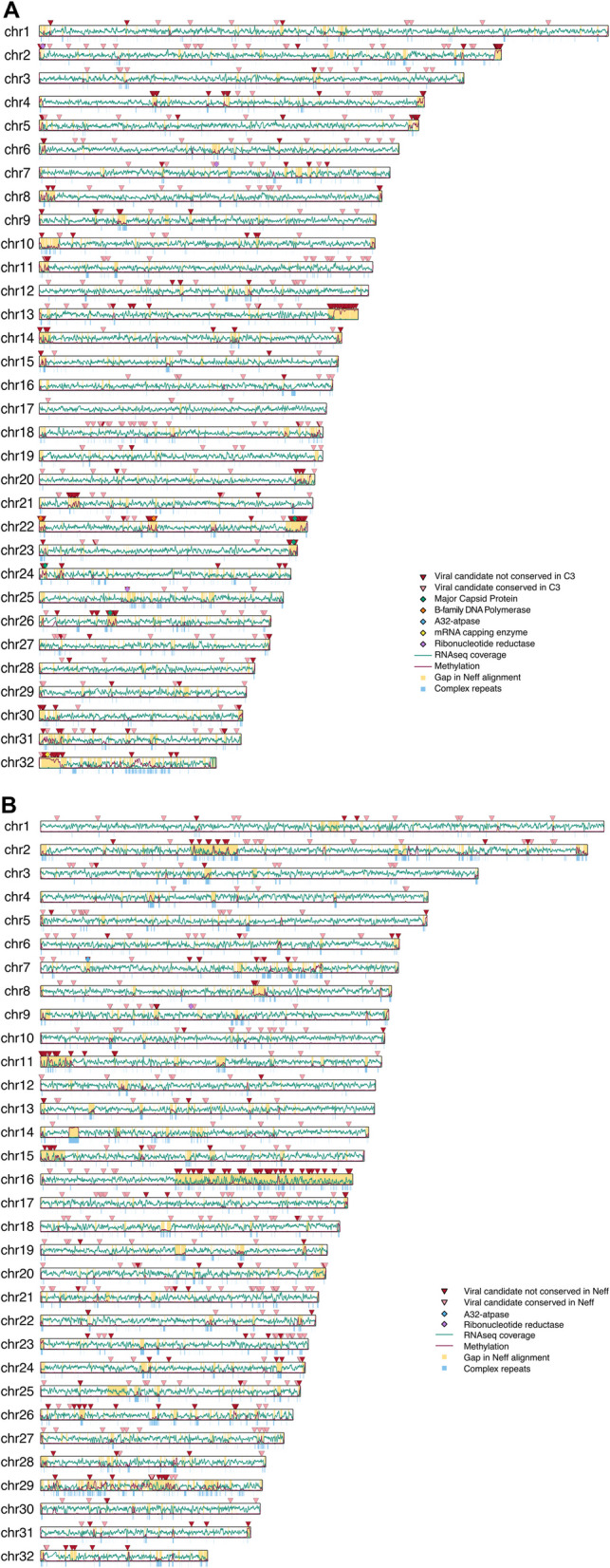
**A** Methylation, expression, and conservation of viral candidates in *Acanthamoeba* str. Neff. The location of conserved (pink arrows) and non-conserved (red arrows) viral candidates as well as viral hallmark genes is mapped across Neff chromosomes 1–32 (2,508,300–777,657 bp), along with the rolling mean of Nanopore-based methylation calls (5,000 bp windows) and log_2_ RNAseq coverage (2,500 bp windows). Regions corresponding to gaps in the Neff-C3 alignment are highlighted in yellow. Complex repeats are mapped in blue below each chromosome. **B** Methylation, expression, and conservation of viral candidates in *Acanthamoeba* str. C3. The location of conserved (pink arrows) and non-conserved (red arrows) viral candidates as well as viral hallmark genes is mapped across C3 chromosomes 1–32 (2,405,921–711,421 bp), along with the rolling mean of Nanopore-based methylation calls (5,000 bp windows) and log_2_ RNAseq coverage (2,500 bp windows). Regions corresponding to gaps in the Neff-C3 alignment are highlighted in yellow. Complex repeats are mapped in blue below each chromosome

We identified 750 viral LGT candidates in Neff and 642 in C3 (Fig. 1A), a substantial increase from the 267 reported by Maumus and Blanc [29] in Neff and the 115 found by Chelkha et al. in *A. polyphaga* [32]. This increase is in large part likely due to improvements in the sampling of giant viruses, as genomic data from new isolates helps with orthologue identification, especially given the large number of ORFans in giant virus genomes. For example, the most common viral best hit in Neff was to *Medusavirus*, a lineage discovered in 2019 [33]. Many of the viral LGT candidates in Neff and C3 are found in viral “islands” (Fig. 2) where they co-occur with orphan genes of similar size and with few introns. Since ORFans are very common in NCLDV genomes [11], we expect that viral homologues will be identified for many of these endogenized sequences as sampling of giant virus genomes continues to improve. Our expanded search criteria also explain some of the increase in identified viral sequences. Our search for viral sequences in intergenic regions included all ORFs 50 amino acids or longer, regardless of whether a start codon could be found. As a result, some groups of viral ORFs are predicted to be pseudogenes, with each ORF mapping to a different segment of the same viral protein. This degraded state suggests that some viral sequences are evolving under relaxed selective constraints. The taxonomic distribution of viral best hits in Neff and C3 is largely consistent, although Neff best hits are dominated by *Medusavirus* whereas C3 best hits are dominated by *Pandoravirus* (Fig. 1A). Phylogenetic trees for integrated major capsid proteins (MCPs) and A32 ATPases, two well conserved viral hallmark genes, show *Medusavirus* nesting within a clade of endogenized *Acanthamoeba* sequences (Fig. 1B, C), with the closely related *Clandestinovirus* as sister group [34]. Better viral sampling might reveal this nesting is due to multiple viral insertions, but it nonetheless strongly supports *Medusavirus* as the true donor of several viral insertions.

### Viral proteins are clustered in poorly conserved sub-telomeric insertions

Putative virus-derived genes in Neff tend to cluster together, forming viral regions usually not conserved in C3 (Fig. 2A). This is in strong contrast to general synteny conservation across both genomes (Fig. S1). This suggests that these viral regions are the product of strain-specific insertions of viral DNA chunks carrying multiple genes. Similar insertions are also found across C3, though they do not appear to have the same density of viral genes as Neff (Fig. 2B). The observed differences in the level of clustering are likely partly influenced by the higher number of lineage-specific viral genes in Neff. Four hundred sixty-eight of the 750 viral candidate proteins in Neff do not have an obvious counterpart in C3, whereas 304 of 642 viral candidate proteins in C3 were not conserved in Neff. Conversely therefore, more viral candidate proteins in C3 were conserved in Neff than vice versa. The discrepancy in the number of conserved regions between Neff and C3 is in part due to the use of nucleotide-level alignment of genomic regions as a proxy for inter-strain conservation. For example, a region encoding a putative viral ORF in Neff might align to a region in C3 of likely viral origin, but where no viral ORF was detected, possibly due to partial deletion of the viral sequence. Likewise, strain-specific duplication of viral regions would cause two regions of one genome to map to only one in the other. These patterns could also be explained in part by secondary loss in Neff and/or C3, but in any case, the lack of conservation of viral DNA insertions between the two strains suggests that they are limited in their taxonomic range.

Only one cluster of viral ORFs is partially conserved between Neff and C3. The cluster is located on one end of Neff chromosome 2 (1–16,471 bp; Fig. 2A), and its counterpart in the C3 genome is a small unplaced scaffold (scaffold_83) that starts with degenerate telomeric repeats. Conserved viral candidates in Neff and C3 otherwise tend to be isolated from other viral candidates in terms of their genomic locations and are usually larger genes with more spliceosomal introns. This is consistent with the hypothesis that most evolutionarily conserved “viral” candidates in *Acanthamoeba* are in fact eukaryotic genes that were acquired by viruses and not vice versa. While those introns could have been acquired after a transfer from viruses, this hypothesis can be excluded for a large number of them. Many conserved viral genes are annotated as serine/threonine kinases and are part of a large family of kinases with a unique domain architecture that is shared between *Acanthamoeba* and giant viruses [28, 29]. Phylogenetic trees of these proteins (Fig. S2) suggest that they have been transferred from nuclear genomes to viruses multiple times, and that their prevalence in the Neff and C3 genomes is more a product of gene duplication common to kinase families [35] rather than multiple recent re-acquisitions.

Viral sequences in Neff are disproportionately found near the ends of chromosome-scale scaffolds and close to both canonical (TTAGGG) and degenerate telomeric repeats. Interestingly, mobile elements and sequences not conserved in Neff and C3 are also disproportionately found in sub-telomeric regions of both *Acanthamoeba* strains (Fig. S3). While sub-telomeric regions in Neff were found to contain numerous mis-assemblies, this spatial bias was observed even after manually curating the Neff scaffolds to remove assembly artifacts. A similar but less pronounced bias can be observed in C3 viral sequences. The difference is likely in part due to the smaller number of lineage-specific viral genes in C3 and to assembly artifacts. Indeed, viral candidates in C3 are disproportionately likely to be found on small scaffolds whose chromosomal location is unclear, at least one of which contains degenerate telomeric repeats. Notably, three of the four largest insertions on chromosome-scale scaffolds in C3 are found directly at the end of a scaffold (see the start of chr11 and chr15 and the second half of chr16 in Fig. 2B).

### The C3 genome harbors a large chimeric region which may have triggered large-scale chromosomal recombination

A striking feature of the C3 viral footprint is the large-scale chimerism of chromosome 16. Two thirds of the chromosome’s 1.3 Mbp sequence, starting at 575 kbp, are not conserved in Neff (Fig. 2B, chr16). The anomalous, non-conserved half of the chromosome is gene poor and enriched for viral candidates, although these are not as densely clustered as in viral insertions in Neff and are interspersed with sequences of apparent eukaryotic origin. Transcription levels are lower and more heterogenous than in the more typical eukaryotic half of the chromosome. Interestingly, a search for viral hallmark genes in this stretch of C3 chromosome 16 using ViralRecall yielded no results. The GC content is unchanged compared to the rest of the genome, and the junction between the “normal” and “chimeric” halves of the chromosome is well supported by long-read data, although multiple breaks in the read coverage can be found starting at ~ 830 kbp. The physical proximity of regions separated by breaks in the long-read coverage is supported by Hi-C data, suggesting that they are part of the same molecule and not the product of an assembly artifact. There is some evidence that this naturally chimeric region has been the catalyst for large-scale chromosomal reorganization, as the non-chimeric half of chromosome 16 in C3 maps to chromosome 2 in Neff. In contrast, chromosome 2 in C3 maps both to the other half of chromosome 2 in Neff as well as Neff chromosome 25 (Fig. S1). While unresolved mis-assemblies prevent us from drawing a definitive conclusion about the organization of C3 chromosome 16, on balance the evidence suggests that it is genuine. *Acanthamoeba* is both highly polyploid and aneuploid [36] and so the association between a massive chimeric region on chromosome 16 and the only large-scale difference between the Neff and C3 assemblies is likely due to the real underlying biology of *Acanthamoeba*.

### Neff and C3 viral proteins have conventional viral functions but insertions in both genomes lack a full suite of viral hallmark genes

Several viral “hallmark” genes, which are highly conserved in NCLDVs and involved in key viral processes, were identified in both genomes. Those include several major capsid proteins, viral polymerase B, and A32 packaging ATPase, among others. Notably, no viral insertion in either genome contained an obvious full suite of viral hallmark genes (Fig. 2), and some hallmark genes (the viral late transcription factor 3 (VLTF3) and superfamily II helicase (SF II)) were missing from all insertions in both Neff and C3. An InterPro search failed to recover a potential function for 380 of the 750 putative viral candidate proteins in Neff. Of those that remained, the most common functional annotation was that of serine/threonine kinase, which is part of a large family of kinases shared with viruses, discussed above. Furthermore, no mobility-associated functions were identified in the viral proteins encoded by either genome, which suggests that these insertions are not capable of mobilizing by themselves in the *Acanthamoeba* genome.

### Viral insertions are dynamic, often chimeric, and sometimes allele-specific

**Fig. 3:**
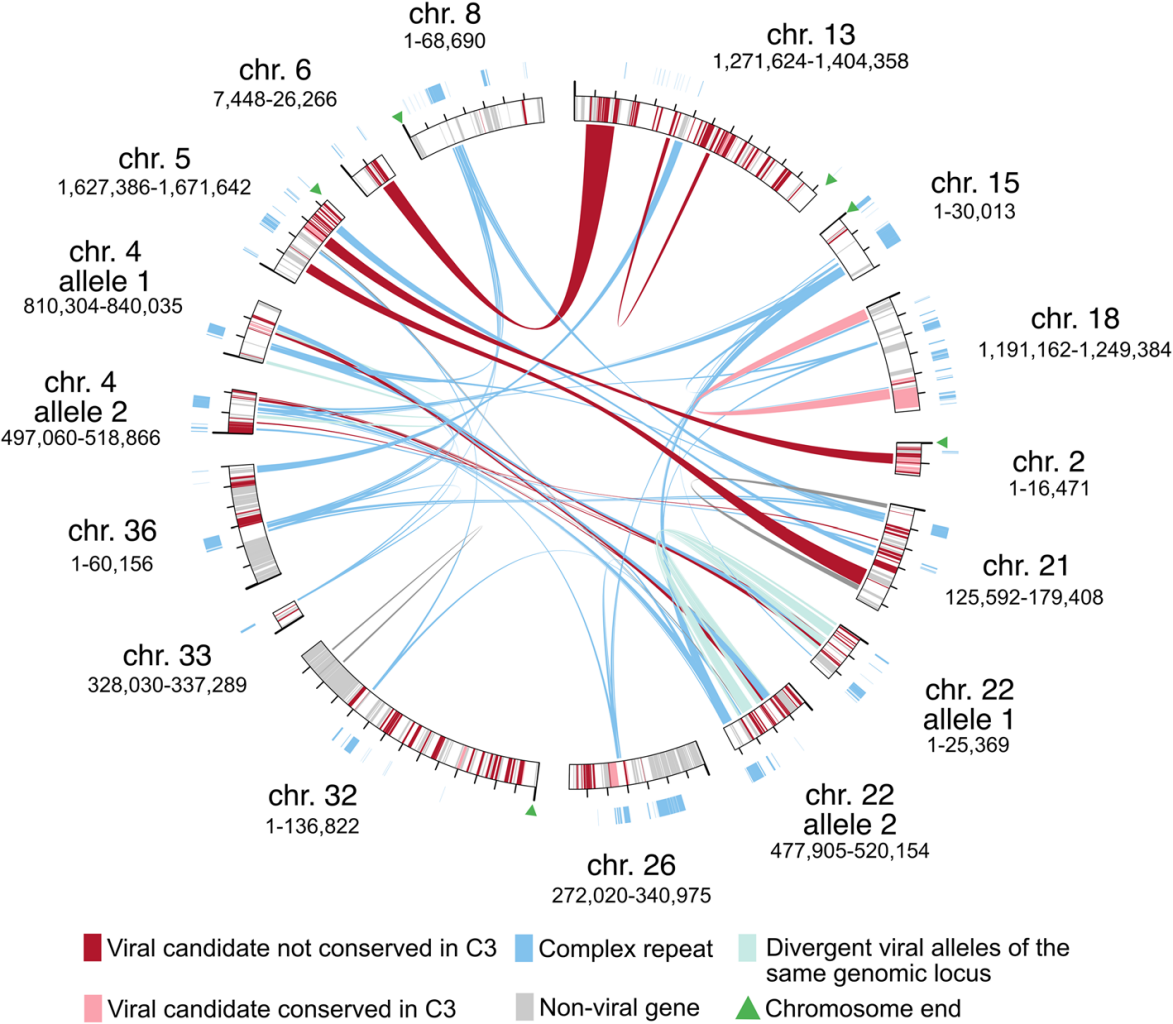
Nucleotide sequence alignment of Neff viral regions, mobile elements, and their genomic context. Circos plot showing nucleotide-level alignments between selected viral regions in Neff. Ticks mark 10 kbp intervals. The start of the sequence is shown by a larger tick. Complex repeats and links between regions containing complex repeats are shown in blue. Viral sequences and links between viral regions not overlapping with complex repeats are shown in red. Alignments between regions which are divergent alleles of the same genomic locus are shown in aquamarine. The genomic location of such alleles was not properly resolved in the Matthey-Doret et al. [30] assembly, causing them to have different genomic coordinates

Genome-scale analysis of viral insertions in *Acanthamoeba* str. Neff speaks to a complex evolutionary history. Sequence alignments of these insertions across different chromosomes show that they share regions with 85–95% nucleotide identity, comparable to the 85% average sequence identity between Neff and C3 (Fig. 3). These duplicated regions range from a few hundred to several thousand nucleotides and typically correspond to a subset of a larger, non-duplicated viral region. In addition to this duplication process, recombination between viral insertions with distinct origins can also be inferred from alignment data. For example, a large sub-telomeric viral region on Neff chromosome 5 appears chimeric, having alignments to distinct viral regions on chromosomes 2 and 21, in addition to being connected to other viral insertions by mobile elements (Fig. 3). Sudden shifts in GC content are also commonplace, supporting the inference of recombination between viral DNAs after genomic insertion (Fig. S4). While duplication or multiple integrations of an already heterogeneous viral insertion followed by differential loss could explain these patterns, post-insertion chimerism is consistent with the observation of transposable elements, duplications, and translocations near and within viral regions.

Multiple viral insertions in the Neff genome are also allele-specific. Allele specificity is inferred when an insertion is robustly supported by some, but not all, reads mapping to the chromosome, indicating the presence of multiple variants of the same position in the genome. Oftentimes the subset of reads that do not map to the viral sequence end in telomeric repeats, indicating that they correspond to polymorphic sub-telomeric regions lacking a viral sequence.

The dynamism of viral regions in Neff cannot be attributed to any intrinsic mobility of viral insertions. As discussed above, no mobility-associated functions were found in InterPro searches for viral candidates, and the possible borders of viral insertions do not obviously correspond to terminal inverted repeats. This, along with the inconsistent size of viral insertions, gene content, and apparent affiliation with giant viruses, speaks strongly against them being transposable elements or polinton-like viruses and virophages.

Instead, duplicated viral sequences in the *Acanthamoeba* str. Neff genome appear to be the product of multiple insertions from giant viruses and/or post-insertion duplications and dispersal events. While it is difficult to know which of these two mechanisms has played the bigger role, the genomic context of viral insertions lends credence to the notion that at least some duplicated viral regions are the product of post-insertion duplication. Viral regions in Neff are both flanked and colonized by mobile elements, elements that are often conserved in C3 even though the surrounding viral sequences are not, indicating that they are native to *Acanthamoeba* and were inserted into viral regions after their endogenization (Fig. S4). These mobile elements form an extensive network connecting otherwise distinct viral regions on multiple chromosomes (Fig. 3). The types of mobile elements observed in Neff and C3 viral regions are largely consistent with those observed within and between genomes. Differences in their distribution across different genomic contexts are nonetheless statistically significant, indicating that viral regions may act as distinct mobile element ecosystems from the rest of the genome (Fig. S5). This association between mobile elements and viral regions reflects a broader trend in *Acanthamoeba*’s genome organization, as both genes for viral proteins and mobile elements are disproportionately found near the ends of chromosomes, which are less conserved than the rest of the genome (Fig. S2). Dynamic changes in the structure of viral insertions in *Acanthamoeba* are thus likely a reflection of the activity of mobile elements native to the genome and of spatial biases in the genome’s organization.

### Viral insertions in the Neff and C3 genomes are transcriptionally suppressed by methylation and heterochromatin

**Fig. 4:**
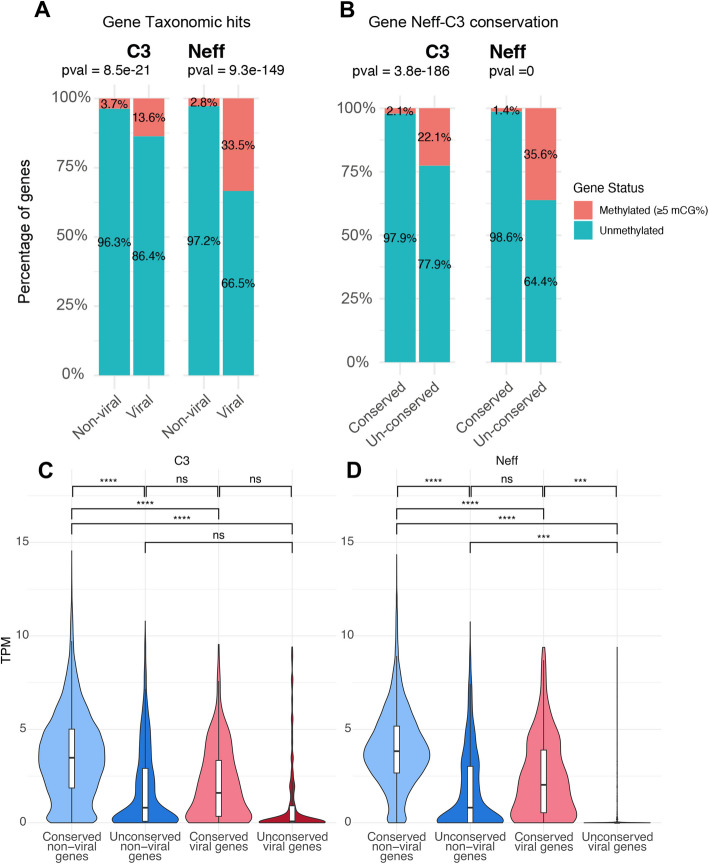
Methylation and expression levels of viral and non-viral genes in *Acanthamoeba* strains. Percentage of methylated and unmethylated genes in Neff and C3, compared between **A** viral and non-viral genes and **B** conserved and non-conserved genes. Methylated genes are defined as genes with ≥ 5% methylation average across all CpGs. Violin and box plot of transcripts per million (log2(TPM + 1)) across gene categories in **C** C3 and **D** Neff, with asterisks indicating significance level for mean differences between pairs of observations

Viral insertions in both Neff and C3 are transcriptionally suppressed. By analyzing 5-methyl-CG signal from nanopore data (from Matthey-Doret et al. [30]), we found that non-conserved regions in general have elevated methylation levels in Neff and C3 (Fig. 4, Fig. S6). In Neff, viral regions in particular were considerably more methylated than other non-conserved regions. In viral regions, intergenic sequences were most heavily methylated, followed by mobile elements, suggesting that suppression of viral DNA is not the only factor at play (Fig. S6). Nonetheless, considerable methylation can be seen in viral genes as well.

**Fig. 5:**
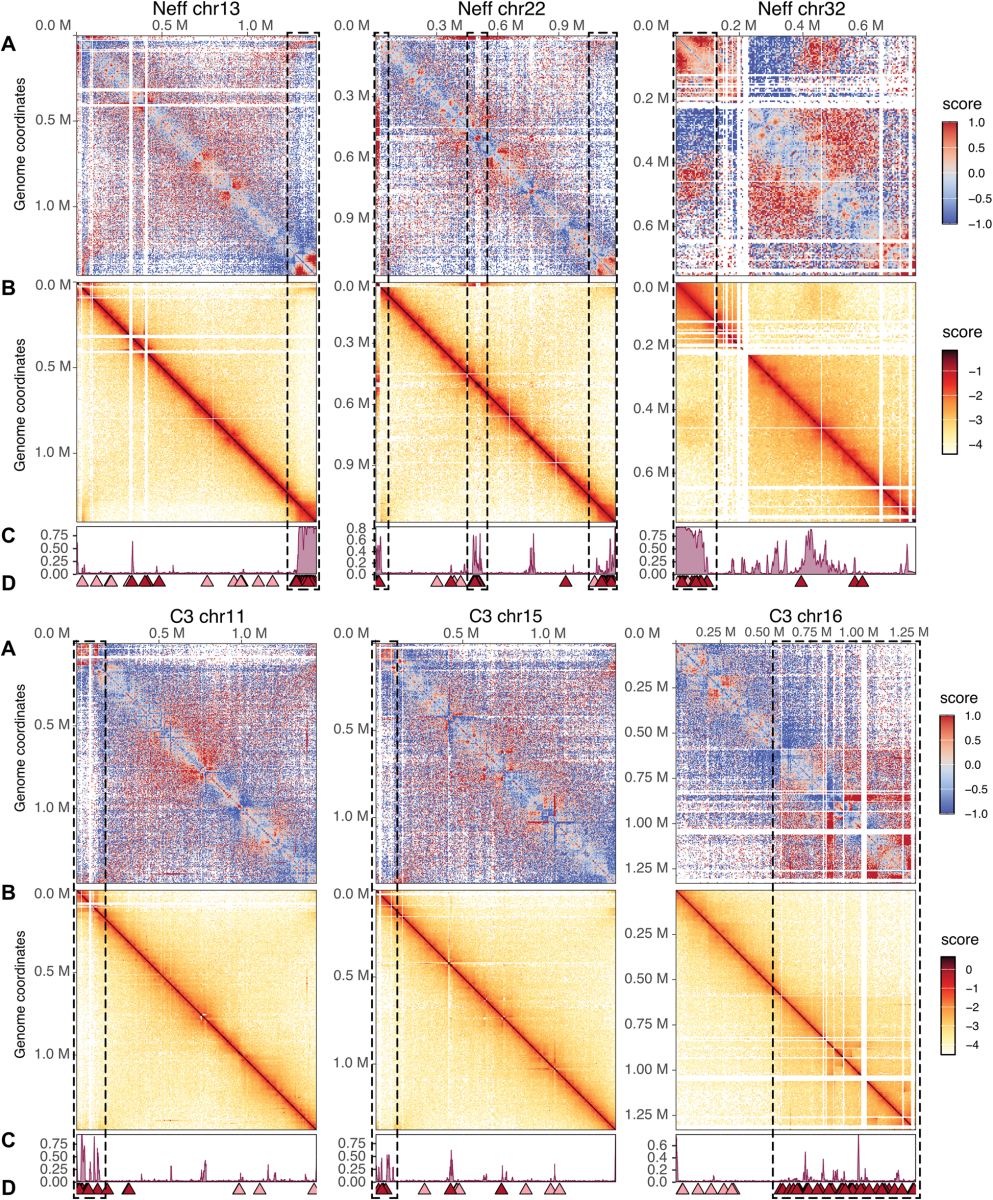
Methylation and DNA packaging of viral insertions in *Acanthamoeba* strains Neff and C3. **A** Detrended chromosome Hi-C contact map of chromosome showing contacts without distance-dependent trends. **B** Chromosome Hi-C contact map. **C** 5mCG level along the chromosome. **D** Location of non-conserved (red) and conserved (pink) viral candidates along the chromosome. Regions corresponding to major viral insertions in **A**, **B**, **C**, and **D** are highlighted by a dotted box

Two viral regions stand out, one at the end of Neff chromosome 13 and the other the beginning of Neff chromosome 32 (Fig. 2). These two regions display homogenous, near-100% methylation levels across their whole length, something not observed anywhere in C3 or elsewhere in Neff. In fact, methylation levels in C3 viral regions are generally lower than those in Neff (Figs. 4, S6, S7) and cover a smaller proportion of divergent regions. Interestingly, in C3, mobile elements account for the most important spike in methylation in divergent and viral regions. Consistent with their elevated methylation, un-conserved viral and non-viral genes in Neff and C3 have considerably lower transcripts per million (TPM) than conserved genes, with un-conserved viral genes having the lowest expression across all genes (Fig. 4A). Hi-C contact maps of the Neff and C3 genomes show that the largest viral insertions correspond to densely packaged chromosome regions, which align with methylation levels across chromosomes (Fig. 5). The genomic location and intensity of methylation is remarkably consistent across technologies (Nanopore and Enzymatic Methyl-seq (EM-seq)) and samples; DNAs extracted from cultures maintained separately over several years showed virtually identical results (Fig. S8). Consistent with these observations, transcription remains low across the length of viral regions (Fig. 2). We conclude that viral regions in *Acanthamoeba* are packaged into stable heterochromatin.

## Discussion

### Viral insertions in Neff and C3 are transient and likely non-functional

We have performed a genome-scale analysis of the origin and fate of viral DNAs inserted into the genomes of two strains of *Acanthamoeba*, an organism that has featured prominently in the field of giant viral research. Our study contributes to a growing body of literature showing that NCLDVs have an extensive but often transitory footprint in protist genomes [20, 22]. Both fragments and full NCLDV genomes have been found integrated in the nuclear genomes of a wide range of unicellular eukaryotes [13, 32, 37]. Despite some ancient and long-lasting contributions of giant viruses to the eukaryotic proteome [21], observed integrations tend to be lineage-specific and vary greatly between lineages and even between closely related species [20]. Our findings are consistent with this pattern. While Neff and C3 were previously thought to be two strains of *Acanthamoeba castellanii*, it has recently been proposed that Neff should be placed with *Acanthamoeba terricola*. Regardless, they have substantial genomic similarity and share 97% 18S rDNA identity, extensive gene synteny (Fig. S1), and ~ 85% of their orthogroups [30]. The average sequence identity of the whole genome alignment is ~ 85%. Despite the high degree of similarity between the two strains, only 38% (282/750) of viral candidates in Neff and 53% (338/642) of viral candidates in C3 are conserved between the two, a large proportion of which are likely bona fide eukaryotic genes identified as viral due to recent host-to-virus transfers. Most viral regions appear to have been acquired after the divergence of the two strains, although we cannot rule out ancestral acquisition and differential loss, the signal for which would be erased due to rapid sequence divergence. The pseudogenization of several viral genes and the observation of recombination events in viral insertions suggests they are evolving under relaxed selective constraints. Furthermore, the active suppression of these viral elements by the cell through methylation and chromatin condensation further buttresses the case that viral integrants are not useful to the cell.

### Viral DNA acquisition in *Acanthamoeba*: endogenization, suppression, and degradation

Whether the widespread presence of viral fragments in protist genomes is a product of accidental or deliberate insertions that are part of the viruses’ life cycle cannot be answered decisively without knowing the identity of the viral donors. As it stands, most viral candidate proteins share low sequence identity to their best viral hit, indicating that closer relatives—whose lifestyles are unknown—may yet be found. We propose a three-step model to explain the widespread discovery of chunks of incomplete viral sequence in the genomes of *Acanthamoeba* and other lineages: (i) integration of viral DNA into the genome, (ii) epigenetic suppression of endogenized viral DNA, and (iii) disruption of the viral DNA by mutational processes.

#### Step 1: nuclear association provides a pathway for viral DNA integration into the host genome

Fragmented viral insertions are observed in a wide range of eukaryotic genomes. Whether these insertions are a product of “accidental” integration during failed infection and/or occur due to endogenization of full viral genomes as part of a latent infection cycle is debatable. In *Acanthamoeba* at least, this question cannot be resolved without knowing the identity of the viral donors. Phylogenies of viral hallmark genes show that *Medusavirus*, or at least some close relative, is a recurrent donor in *Acanthamoeba*. BLAST best hits suggest *Pandoravirus* is also a common donor, with a virus closely related to *Mollivirus sibericum* having contributed at least one MCP gene as well (Fig. 1). The same *Medusavirus* and *Mollivirus* MCPs were also found in Neff by Willemsen et al. [38].

NCLDV infection is a highly disruptive process. In the case of *Mimivirus*, the first discovered giant virus [24], which is known to infect *Acanthamoeba*, the cell nucleus shrinks dramatically during infection even though viral replication occurs entirely in a cytoplasmic viral factory [39]. *Pandoravirus*, on the other hand, requires entry of the viral genome into the nucleus for replication, followed by the formation of a viral factory in the cytoplasm and degeneration of the nuclear membrane [39]. *Medusavirus* replicates inside of the host nucleus and appears to leave the nucleus morphologically intact until later in the infection process, which often results in encystment of the cell [33]. Given these observations, close contact with and disruption of the *Acanthamoeba* nucleus may result in accidental integration of viral DNA fragments into the nuclear genome. It is conceivable that yet to be discovered non-lytic close relatives of *Medusavirus* and *Pandoravirus* could also have colonized the *Acanthamoeba* genome. While the majority of known NCLDVs are lytic, current NCLDV sampling methods rely on the observation of cell lysis [14] and as such bias discovery towards the most lethal viruses. That said, our phylogenetic trees suggest that this would have to be a close relative of the lytic *Medusavirus*. Either way, the identification of endogenized viruses also related to *Medusavirus* in the distantly related opisthokont *Amoebidium* [22] supports the case that the Mamonoviridae lineage or its close allies are prolific viral DNA donors.

#### Step 2: suppression allows viral DNAs to remain in the genome over evolutionary timescales

The pervasiveness of viral methylation in *Acanthamoeba* raises questions about the nature of host-virus interactions. Viral regions in Neff are more heavily methylated than other divergent regions. This could suggest that NCLDV integration represents a common enough threat to warrant a dedicated cellular response, buttressing the case that integrations are degenerate products of endogenized, latent viruses. This is not necessarily the case, however. Viral regions in Neff are more heavily methylated than other non-viral regions divergent from Neff, but mobile elements and intergenic DNA in these regions appear even more highly methylated than viral genes. Furthermore, mobile elements in C3 show greater levels of methylation than viral genes. Hypermethylation of viral insertions could thus be a byproduct of more generic self/non-self defense systems targeting any foreign DNA in *Acanthamoeba*, which would affect even rare accidental integration events.

Whether viral DNA integration in *Acanthamoeba* is accidental or not, its silencing by 5-methyl-CG base modification likely facilitates the persistence of viral insertions over evolutionary timeframes. If viral DNA enters the genome because of an endogenous “lifestyle,” silencing it will reduce the likelihood that the virus kills the cell. In this way, suppression reduces the fitness cost of the inserted virus while also allowing it to persist in the genome over multiple generations. These fitness benefits also likely apply in cases of accidental integration. Viral infection results in substantial host cell reorganization; *Pandoravirus* recycles the nuclear membrane for virion formation [40], diverse NCLDVs encode a wide range of metabolic genes to reprogram the infected host cell [41], and even cellular locomotion can be altered, as the *Mimivirus* relative *Tupanvirus* induces infected amoebae to seek out and aggregate with non-infected cells [42]. The expression of even an incomplete viral proteome could impact host fitness, a fitness cost which methylation reduces regardless of whether it evolved specifically to target viruses. In this scenario, the widespread distribution of virus insertions could be a mere byproduct of the reduced fitness cost of rare accidental insertions, thanks to non-specific methylation of foreign DNA.

Our results are broadly consistent with work on the opisthokont *Amoebidium*, where large hypermethylated islands coincided with partial NCLDV integrations. Moreover, other unicellular holozoans showed a correlation between 5-methylcytosine modifications and the presence of giant virus endogenizations [22]. Similarly, giant virus endogenizations were found to be methylated in the moss *Physcomitrium patens* [43]. 5-Methyl-CG appears to enable the co-evolution of selfish DNA with the genome by reducing the fitness cost of genetic parasites. Our account of hypermethylated giant virus insertions in *Acanthamoeba* strengthens the applicability of this model to *Amoebozoa* and shows that methylation enables similar patterns of integrations in distantly related organisms.

#### Step 3: degradation neutralizes the virus and produces observed patterns of scattered viral insertions

Host-associated molecular silencing mechanisms that limit translocation and mobile element spread facilitate the final step in the inserted DNA’s evolutionary trajectory. Once silenced, the viral DNAs are at the mercy of mutational processes which will mutate and re-arrange them, inactivating any complete viral genomes and degrading individual protein-coding sequences before eventually deleting them. These processes are on display in the *Acanthamoeba* str. Neff genome, where partial copies of viral insertions are found scattered throughout the genome. Examples of deletion and recombination can readily be observed across duplicated viral regions, as can be colonization of viral insertions by transposable elements from the host genome. Once broken down as such, the virus seems far less likely to have a fitness effect on the cell, which nonetheless continues to repress it. The sub-telomeric bias of viral integrations in Neff reflects broader genome-wide patterns in genome organization; sub-telomeric regions are generally less conserved, gene poor, and enriched in mobile elements relative to the rest of chromosomes. Integrated viral DNAs are thus subject to broader genomic forces shaping the *Acanthamoeba* genome.

Across eukaryotes, sub-telomeric regions have been linked to relaxed selection, lateral gene transfer, and transposable elements [44–46], making them both plausible landing pads for viral DNA and obvious sites for duplication and deletion. Likewise, the mobile elements found near viral regions may act as vectors of mobility, either by dragging along adjacent viral sequences during transposition or by mediating homologous recombination events. Interestingly, Matthey-Doret et al. [30] found that telomeres tend to cluster together in *Acanthamoeba*, and heterochromatin compartments are often found packaged together near the nuclear membrane [47]. This is consistent with the hypothesis that the sub-telomeric features of *Acanthamoeba* are linked to the higher-order physical organization of DNA in the genome.

Ironically, the same processes that neutralize viral DNA over extended periods of time provide an opportunity for viral genes to very occasionally become functional. The translocation of DNA throughout the genome could sometimes allow viral genes to acquire appropriate transcription regulatory sequences and become expressed, and their integration in specific alleles may allow the healthy, original allele(s) to compensate for potential deleterious effects, providing a window of time for neofunctionalization. But successful acquisitions are overwhelmingly exceptions, judging from current patterns of virus-to-eukaryote transfers [12].

### Broader implications across the tree of eukaryotes

How applicable is this three-step model across the eukaryotic tree of life? One major obstacle to generalization is the difficulty of comparing different genomic assemblies. Even between *Acanthamoeba* strains Neff and C3, we find divergences. Viral insertions in the C3 genome are less numerous and less clearly biased towards sub-telomeric regions as in Neff. The extent to which these differences are an artifact of assembly problems linked to imperfect long-read sequence data and aneuploidy remains to be seen. Viral insertions in *Acanthamoeba* can be biologically complex and consequentially vulnerable to mis-assembly, and in strain C3 viral sequences are disproportionately found on small scaffolds. It is, however, harder to dismiss as artifactual the striking difference in methylation levels between Neff and C3. Methylation levels in divergent regions in Neff are markedly higher than those in C3 and spread out between mobile elements, intergenic sequences, and viral DNA, whereas in C3 mobile elements are far more targeted. The impact of these differences in selfish DNA suppression strategies on the evolutionary trajectory of *Acanthamoeba* remains to be determined.

Some clear general patterns can nevertheless be observed across genomic data. Complete, partial, and highly degraded viral genomes have all been found integrated in the genomes of diverse protists, indicating that there exists a spectrum of viral integration—from mirusvirus elements in *Aurantiochytrium* and other thraustochytrids [16] to the “giant endogenous viral elements” of the green alga *Chlamydomonas* [48] and brown alga *Ectocarpus* [49], to the scattershot integration described here in *Acanthamoeba*. Hypermethylation of fragmented viral regions has also been observed in the unicellular opisthokont *Amoebidium* [22] which also shows evidence for recurrent, transient viral integration. While the model we outline is by no means the only existing pathway to integration, evidence suggests that it is likely very common. Understanding the interplay between methylation and integration will be particularly important to tease out variations in this model, thereby revealing differences in the way that epigenetics is likely to be utilized as a “tool” by eukaryotic hosts to regulate the expression of foreign DNA in particular and resident genes in general.

We have shown that viral DNA insertions in *Acanthamoeba* genomes are numerous but transient, and do not contribute to the kind of genome bloat seen in many complex multicellular organisms or in dinoflagellates. The value, or lack thereof, that selfish DNAs such as those coming from viruses brings to their host is a long-standing debate. As the study of viral integration in protists comes to maturity, it becomes possible to recast debates around selfish DNA in a truly phylogenetically and comparatively rigorous context. Cells everywhere are constantly exposed to a barrage of foreign DNA. How they respond to it, and what they do with it, is a fundamental question for our understanding of genome evolution.

## Conclusions

We analyzed chromosome-scale assemblies for the *Acanthamoeba* strains Neff and C3 and identified hundreds of new potentially viral sequences. Our analysis shows that viral insertions are largely transient and not conserved between the two genomes. Most conserved viral candidates are in fact likely eukaryotic genes which happen to have viral homologues due to eukaryote-to-virus LGT. Viral insertions correspond to partial chunks of larger viruses and are highly methylated and packaged into heterochromatin. Multiple partial copies of viral insertions can be found throughout the genome, which may result from multiple insertions by the same virus or duplications following insertion. Although they are not mobile, the position of viral DNA fragments in the genome is likely dynamic. This dynamism is multifaceted and includes duplication of subsets of viral regions, chimeric regions combining multiple viral insertions, host-mobile element colonization of viral regions, and allele-specific variants of viral integrations. We synthesize these observations into a cohesive integration, suppression, and degradation model of the viral footprint in *Acanthamoeba*, which can be applied to and tested in other lineages. Integration may result from accidental recombination between the viral and host genomes during viral replication in the nucleus, and/or from complete endogenization of viral genomes into the host. Suppression further explains how the cell survives the initial infection and can retain the virus over multiple generations. Degradation permanently inactivates the viral DNA, shattering it into smaller pieces which, although they do not encode machinery for their own mobility, can be dispersed throughout the genome by mutational processes intrinsic to the host. Observed viral integrations represent transitory stages between insertion and deletion. Viral DNA in *Acanthamoeba* is thus likely the evolutionary byproduct of recurrent close contact with viruses and does not appear to have long-term effects on genome content, although it may facilitate intra- and inter-chromosomal recombination events. Why some genomes appear to retain the large amounts of “junk” they receive while others, like *Acanthamoeba*, delete them relatively quickly over evolutionary timescales is a question that still needs to be addressed.

## Methods

### Detection of viral sequences

Genome assemblies and gene models for *Acanthamoeba* strains C3 and Neff used in this study were from Matthey-Doret et al. [30]. ORFs 150 nucleotides and longer were extracted from intergenic regions using ORFm [50] and were not required to have a start codon. Predicted proteins and intergenic ORFs for Neff and C3 were BLASTed against a local copy of the NCBI nr database (downloaded May 2024) using Diamond BLAST [51] with an *e* value cutoff of 0.001. All hits to the *Acanthamoeba* genus were filtered out to avoid missing recent LGTs. Protein sequences with a best hit to viruses, or which had viruses as half or more of their top ten hits, were retained as viral candidates and mapped to the Neff and C3 assemblies. ViralRecall was also used to cross-reference our viral candidates [52].

Giant virus markers identified with ViralRecall were used to build phylogenetic trees using GVDB as a backbone [34]. All major capsid proteins (MCPs) and A32 ATPases encoded in highly contiguous *Acanthamoeba* assemblies [30, 38] were used as queries for BLASTp searches against GVDB. The top 20 hits were retained for each sequence and complemented with newer sequences from the NCBI NR database. Multiple sequence alignments were generated using MAFFT with L-INS-I mode, the resulting alignments were trimmed using trimAl, and maximum likelihood trees were built using IQ-TREE 2, allowing for automatic model testing and 1000 ultrafast bootstrap and aLRT as nodal support values.

### Analysis of genome conservation between Neff and C3

A whole genome alignment of the Neff and C3 assemblies was performed using MUMMER4 [53] with an extend value of 1000 bp to avoid losing alignments due to short gaps. Candidate viral proteins were identified as being conserved between the two genomes if they mapped wholly or partially to regions that could be confidently aligned between the Neff and C3 genomes. They were identified as not conserved if they completely overlapped with a gap in the alignment. The show-diff function was used to identify differences between the Neff and C3 assemblies and identify possible duplications and translocations. The boundaries of viral integrations were sometimes difficult to identify, as gaps in the Neff-C3 alignment corresponding to viral ORFs were often interrupted by duplications and/or translocations from elsewhere in the genome. A custom script was written which concatenated together contiguous gaps, translocations, and duplications. Divergent regions obtained using this script were categorized as “viral regions” if at least one full viral gene was found within them. To avoid including obviously eukaryotic regions in these viral regions, coordinates were further manually curated based on GC content shifts, gene density, and the taxonomy of BLAST best hits of neighboring genes. In parallel, one to one orthologues between Neff and C3 assemblies were identified using OrthoFinder, and these were used as input for MacroSyntR [54] to represent chromosome homology and synteny conservation.

### Comparison of viral insertions within the Neff and C3 genomes

All concatenated divergent nucleotide regions coding for putative viral proteins in Neff were BLASTed [55] against one another, using the DUST program to filter out hits to low-complexity sequences. A detailed alignment was then performed for selected viral insertions using Sibelia [56] and Circos [57].

### Functional annotation and detection of mobile elements

Predicted functions were assigned to all Neff and C3 predicted proteins and intergenic ORFs using an InterProScan [58] search with an *e* value cutoff of 0.001. To further analyze the completeness of lineage-specific viral insertions, viral regions in the Neff and C3 genomes were searched for viral hallmark genes using ViralRecall. Hits with an *e* value below 1 × 10^−5^ and which were found in a viral region were retained. The InterProScan annotations for viral candidates were manually examined for functions indicative of mobility. Mobile elements and simple repeats in Neff and C3 were predicted using RepeatMasker [59], and differences in mobile element content between different genomic regions were calculated using a chi-square test.

### Expression and repression of viral sequences

RNA-Seq reads from strain C3 (uninfected with *Legionella*) from Matthey-Doret et al. [30] (GenBank SRR15928348, SRR15928350, SRR15928349) [60] were combined into one file and aligned to the C3 genome assembly using HISAT2 [61]. Neff RNA-Seq reads (GenBank SRR11193414) [62] were similarly aligned to the Neff assembly and the log_2_ value of coverage across both assemblies were calculated. Transcript per million values were calculated with Kallisto [63] for all predicted proteins and viral intergenic ORFs using sequences from SRR15928348 for C3 and SRR11193414 for Neff. *T*-tests were performed to confirm the statistical significance of differences in expression levels. Hi-C data generated by Matthey-Doret et al. [30] was recovered from https://zenodo.org/records/6800059 (31/10/2024) [64] to identify chromosome territories and patterns of DNA packaging associated with viral insertions. 5mCG base modifications in Neff and C3 were predicted by running the Nanopolish call-methylation package [65] on the raw fast5 reads underpinning each assembly. The reliability of Nanopolish 5mCG identification was established by comparing results to Enzymatic Methyl-seq data generated by Sarre et al. [66] (Fig. S8). In both cases, methylation level was defined as the percentage of methylated reads aligning to a CpG site. Genome-wide methylation patterns were visualized using a rolling mean of methylation levels across the genome. For more targeted analysis, sequences with an average of ≥ 5% methylation across all CpG sites were counted as methylated and *t*-tests were performed to compare the statistical significance of differences in methylation. Neff nanopore fast5 files were generated in the Archibald Lab and the nanopore fast5 files for C3 were provided by Cyril Matthey-Doret, stemming from Matthey-Doret et al. [30].

### Controlling for mis-assemblies and allele-specific variants

Mis-assemblies can be a major confounding factor when analyzing spatial distribution patterns of viral DNAs in nuclear genomes. To control for this fact, nanopore long reads from both the Neff and C3 assemblies were mapped against the final assemblies using minimap2, and Hi-C contact data generated by Matthey-Doret et al. [30] were considered for all chromosome-scale scaffolds. For the Neff assembly, we manually inspected both the long read alignment and the Hi-C plots to identify possible mis-assemblies. Genomic regions were considered to be possible mis-assemblies if a sequence’s location lacked both long read mapping and Hi-C support. Spatial patterns in the distribution of Neff viral genes and translocations were filtered to exclude sequences falling within these compromised regions and reanalyzed to show that observed biases still held. In several cases, mis-assemblies appeared to be a product of allele-specific variants not being properly reflected in the assembly and in some cases artifactually grafted onto the ends of scaffolds. The true location of these variants could be resolved using Hi-C data and by mapping reads to their true location.

## Supplementary Information

Additional file 1. Figures S1–S8. Word document. Fig. S1 Synteny between Neff and C3 chromosomes. Fig. S2 Phylogeny of serine/threonine kinase endogenized into the Neff genome. Fig. S3 Distance from chromosome ends of genomic features in Neff and C3. Fig. S4 Detailed characterization of representative viral insertions in Acanthamoeba str. Neff. Fig. S5 Mobile element diversity based on genomic context in Neff and C3. Fig. S6 Methylation level of genes, mobile elements, and intergenic regions in Acanthamoeba strains Neff and C3. Fig. S7 Methylation level of viral genes and regions in Acanthamoeba strains Neff and C3. Fig. S8 Comparison of Nanopolish and Enzymatic Methyl-seq analyses.

## Data Availability

All data generated or analysed during this study are included in this published article, its supplementary information files and publicly available repositories. Genomic, transcriptomic and Hi-C data analyzed in this study are from Matthey-Doret, Colp et al.30,60,62 All data and code on which the conclusions of this study rely are available on Zenodo (10.5281/zenodo.15492747) [67].

## Abbreviations

EM-seq: Enzymatic Methyl-seq
GEVE: Giant endogenized viral element
kbp: Kilobase-pair
TPM: Transcripts per million
LGT: Lateral gene transfer
Mbp: Megabase-pair
MCP: Major capsid protein
5mCG: 5-Methyl-CG
