# Supplementary material for: Epigenetic silencing and genome dynamics determine the fate of giant virus endogenizations in Acanthamoeba
Source: BMC Biol. 2025 Jul 1;23:171. doi: 10.1186/s12915-025-02280-1 (PMC12210455; doi:10.1186/s12915-025-02280-1)
Supplement: Supplementary file 1 — Additional file 1. Figures S1–S8. Word document. Fig. S1 Synteny between Neff and C3 chromosomes. Fig. S2 Phylogeny of serine/threonine kinase endogenized into the Neff genome. Fig. S3 Distance from chromosome ends of genomic features in Neff and C3. Fig. S4 Detailed characterization of representative viral insertions in Acanthamoeba str. Neff. Fig. S5 Mobile element diversity based on genomic context in Neff and C3. Fig. S6 Methylation level of genes, mobile elements, and intergenic regions in Acanthamoeba strains Neff and C3. Fig. S7 Methylation level of viral genes and regions in Acanthamoeba strains Neff and C3. Fig. S8 Comparison of Nanopolish and Enzymatic Methyl-seq analyses. [file 12915_2025_2280_MOESM1_ESM.docx]

**SUPPLEMENTARY FIGURES**

**
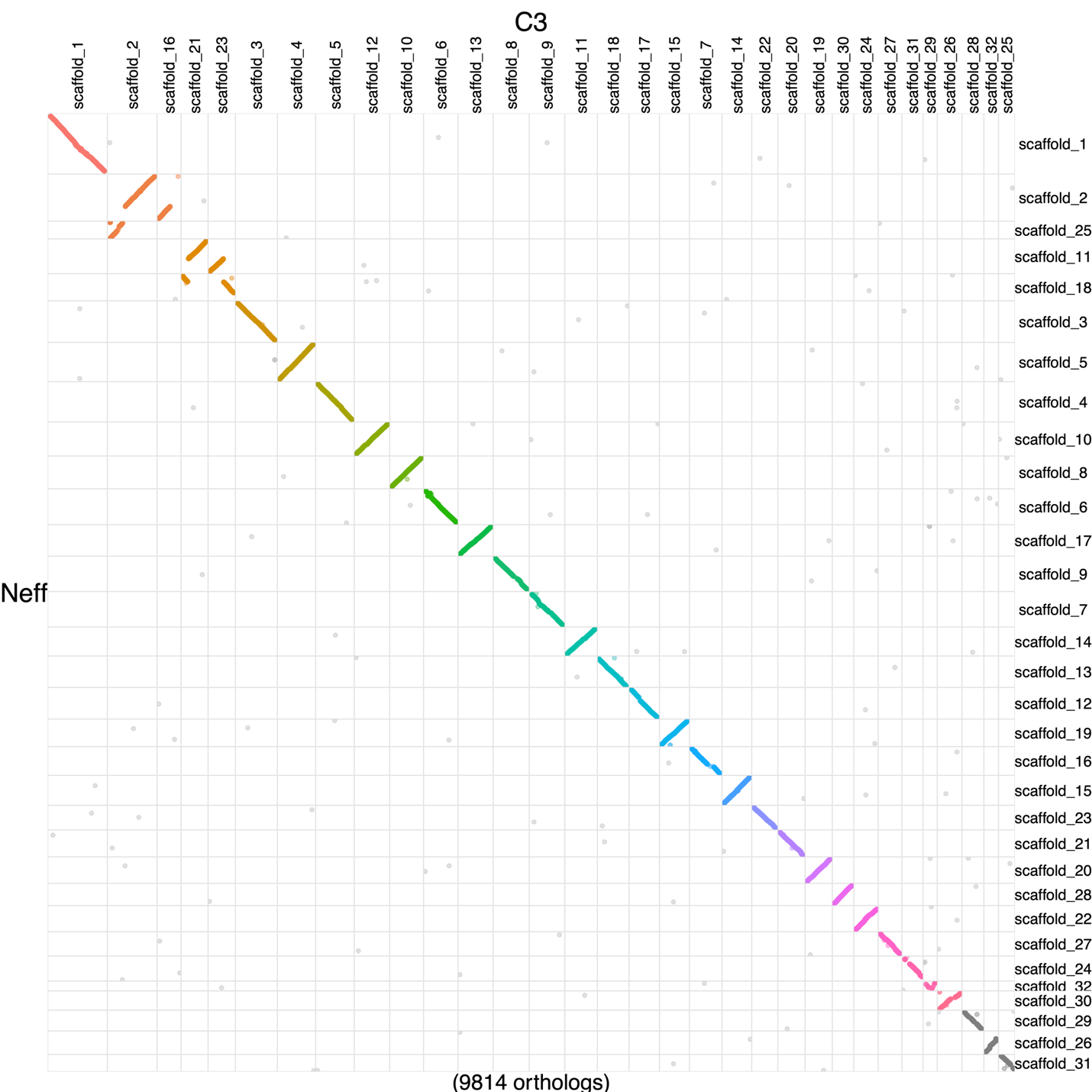
**

**Supplementary Figure S1: Synteny between Neff and C3 chromosomes.** Dot-plot of genome wide synteny between orthologues in Neff and C3 produced using macrosyntR.

**
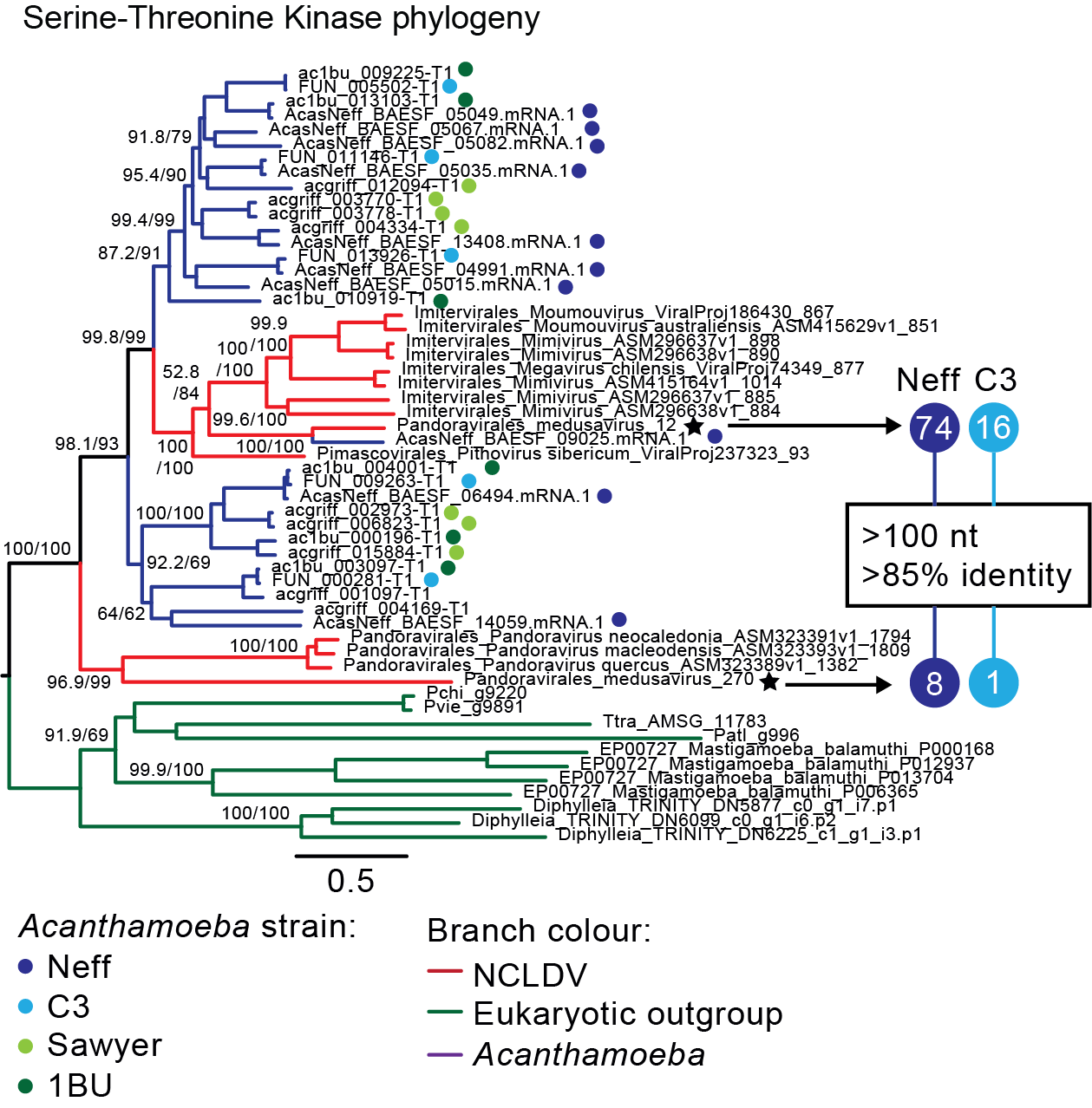
**

**Supplementary Figure S2: Phylogeny of Serine/Threonine Kinase endogenized into the Neff genome.** Phylogeny of Serine/Threonine Kinases endogenized into the Neff genome. Circles indicate the number of BLASTn hits using the *Medusavirus* Serine Threonine Kinase sequences against the Neff and C3 genomes, with the total amount of hits displaying >85% identity to the *Medusavirus* sequence, and at least 100 nt in length. The model was auto-selected by IQ-TREE 2.

**
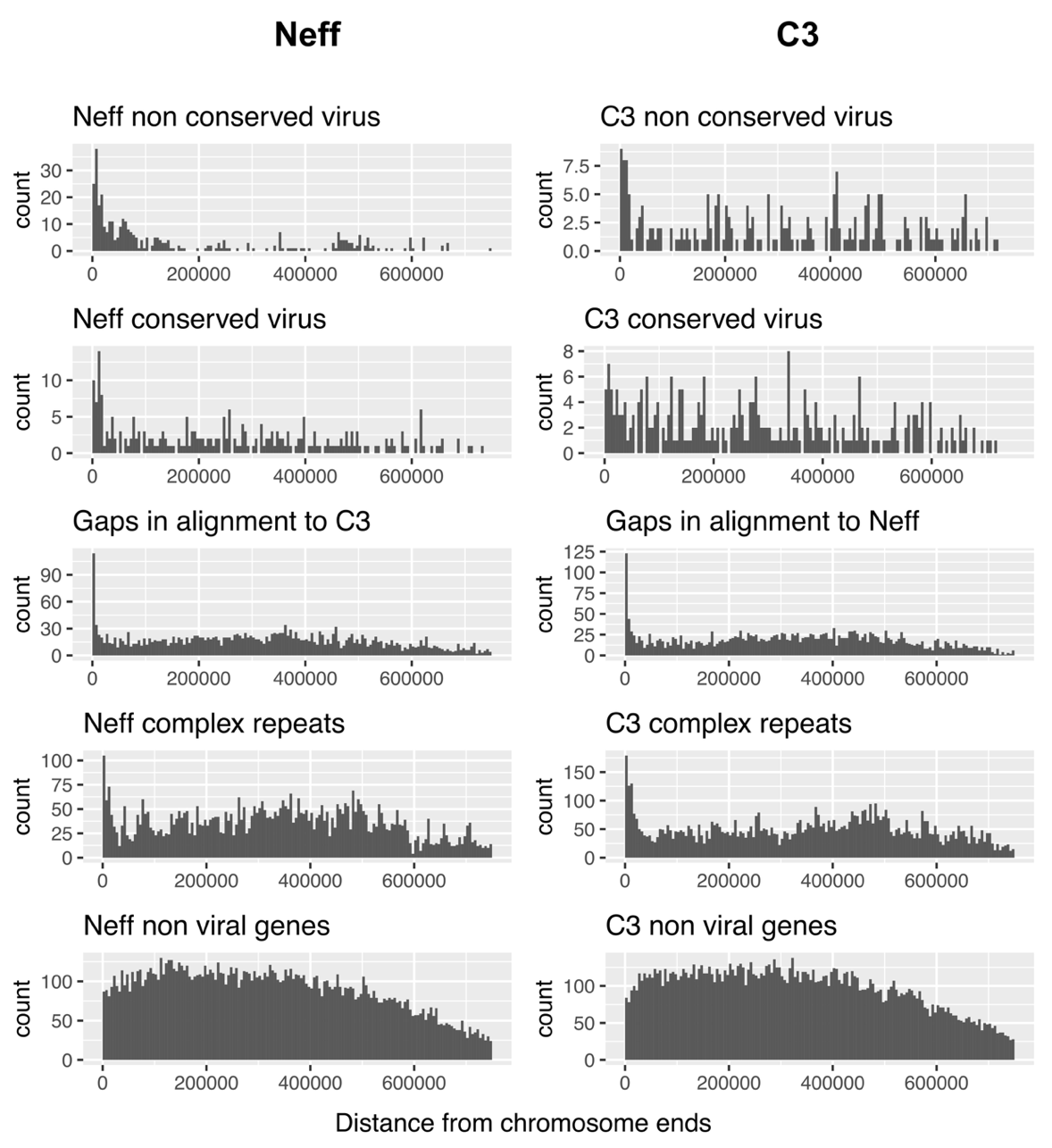
**

**Supplementary Figure S3: Distance from chromosome ends of genomic features in Neff and C3.** The distance of each viral candidate, RepeatMasker complex repeat, non-viral gene, and gap in the Neff-C3 alignment, from the end of their respective chromosome was calculated. Only features found on chromosome scale scaffolds (scaffolds 1-32) were included. Neff genomic features were filtered to correct for errors in position due to misasemblies. Values were binned into 5,000bp increments to make a histogram of distance from chromosome ends, showing trends in the spatial organization of different genomic features across chromosomes.

**
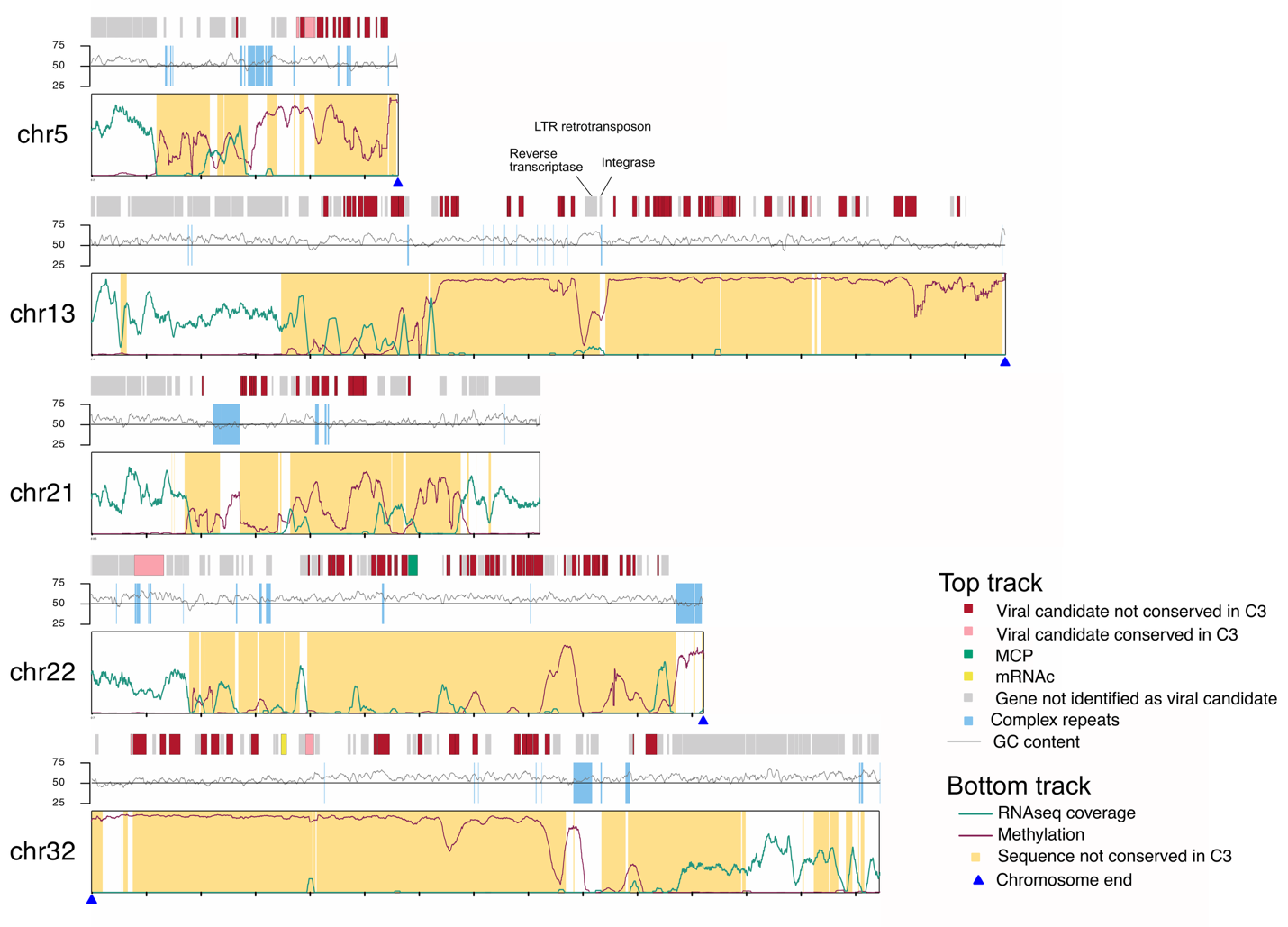
**

**Supplementary Figure S4: Detailed characterization of representative viral insertions in *Acanthamoeba* str. Neff.** Methylation levels were graphed using a 1500bp rolling mean. GC content was graphed using a 500bp rolling mean. The log_2_ value of RNAseq coverage was graphed using a 1000kp rolling mean. Viral hallmark genes, viral candidates and genes not identified as viral candidates are mapped above each sequence.

**
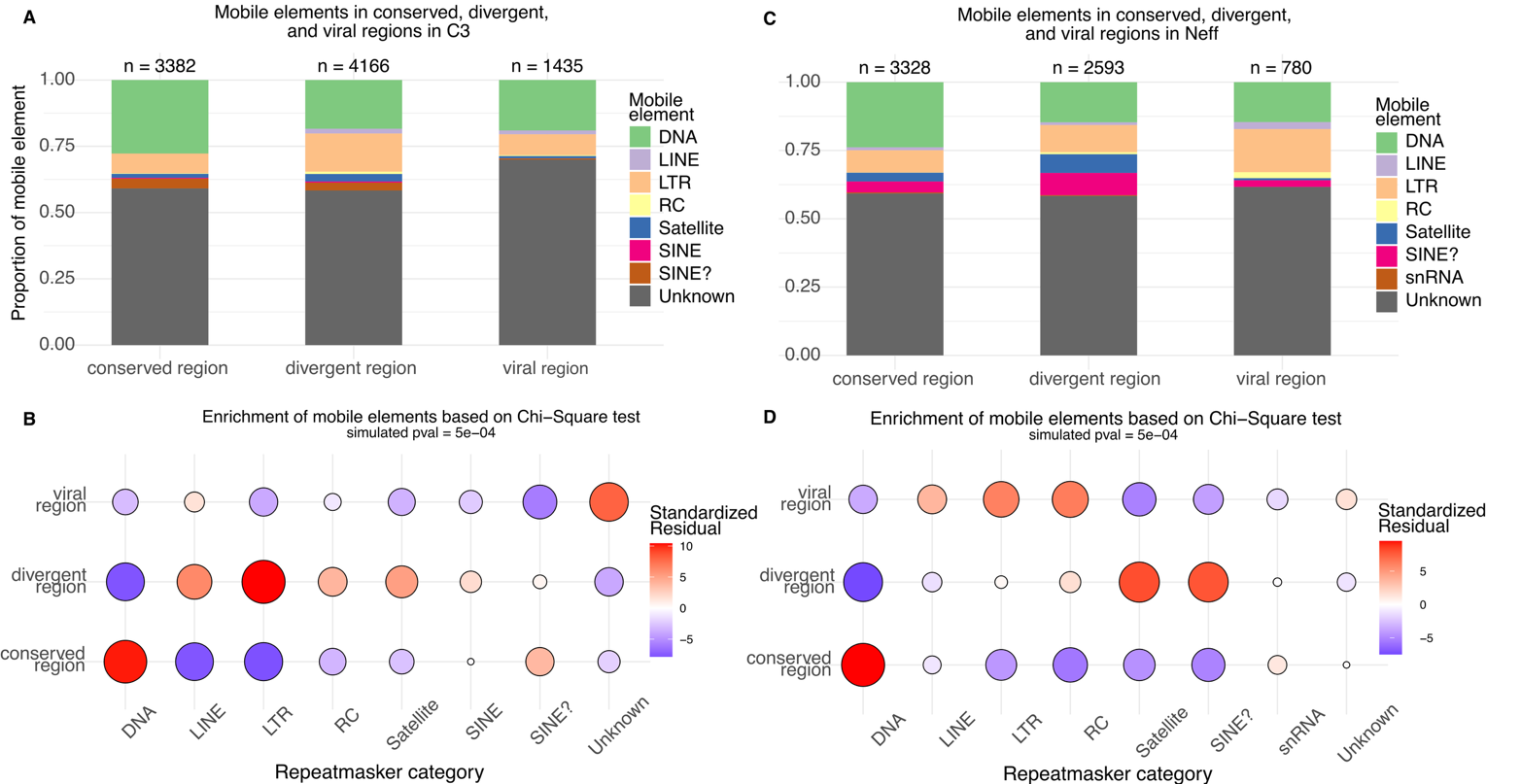
**

**Fig. S5 Mobile element diversity based on genomic context in Neff and C3.** Type of complex repeats in regions conserved between Neff **(A)** and C3 **(C)** in regions conserved between the two genomes, divergent regions lacking any complete viral candidate, and viral regions. A chi-square test found that the differences between genomic contexts were statistically significant in both Neff and C3 (simulated p-value 5e-04 in both cases). **(B)** Bubble plot showing standardized residual for each pair of categories, indicating the relative contribution of each category to the p-value (Neff **(B)**, C3 **(D)**).


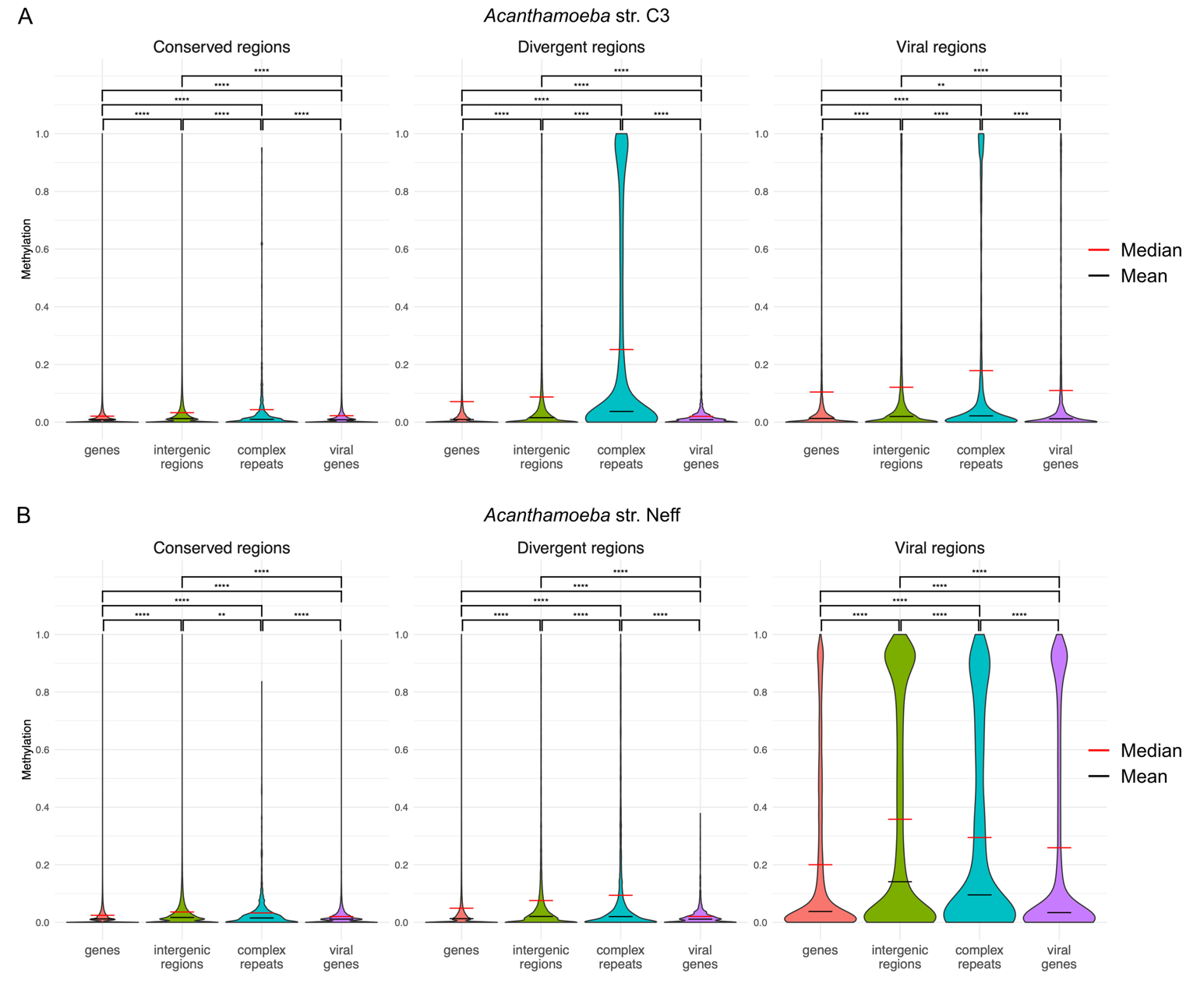


**Fig. S6 Methylation level of genes, mobile elements and intergenic regions in *Acanthamoeba* strains Neff and C3.** Violin plot of the methylation level of every CpG site across genes, intergenic regions, complex repeats predicted by RepeatMasker, and viral candidates in C3 **(A)** and Neff **(B)**. Plots are shown for regions conserved between Neff and C3, divergent regions between the two genomes lacking any complete viral candidate, and viral regions. The median and mean of each distribution is shown. Asterisks indicate significance level for t-tests between pairs of observations.


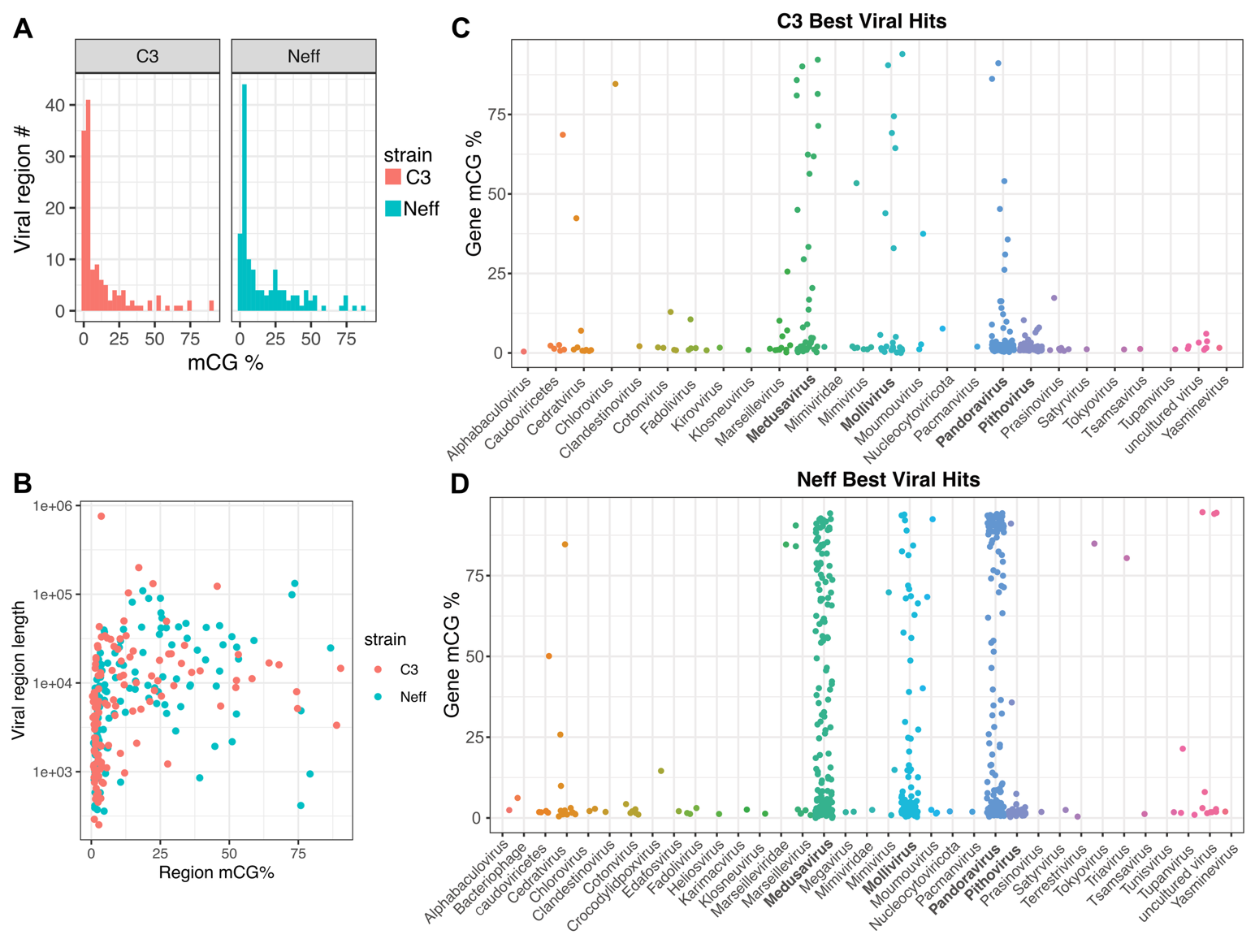


**Fig. S7 Methylation level of viral genes and regions in *Acanthamoeba* strains Neff and C3.** Histogram of methylation rates across viral regions **(A)**. Scatterplot of methylation rate and viral region length **(B)**. Methylation rate of viral genes by taxonomy in C3 **(C)** and Neff **(D)**. Methylation rate is defined as the average mCG% of all CpG sites over a given gene or region.


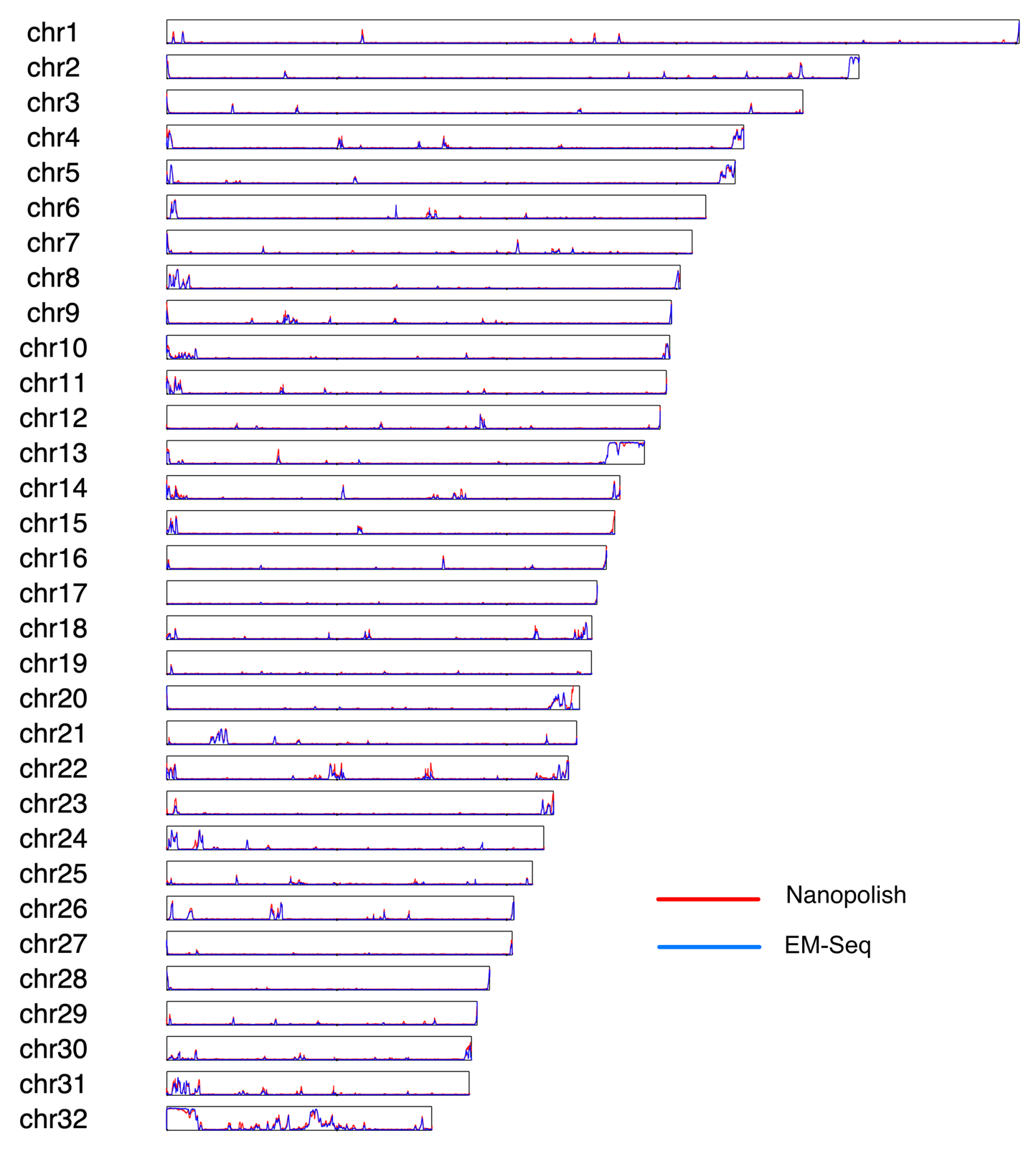


**Supplementary Figure S8: Comparison of Nanopolish and Enzymatic Methyl-seq analyses.** A 5,000 bp rolling mean of the methylation percentage is mapped across each Neff chromosome for both Nanopolish-based methylation calls (red) and Enzymatic Methyl-seq data (blue).
